# Blb-NRF2-PON1 Cross-Talk in Abdominal Aortic Aneurysm Progression

**DOI:** 10.3390/antiox12081568

**Published:** 2023-08-05

**Authors:** Magdalena P. Kasprzak, Bogna Gryszczyńska, Anna Olasińska-Wiśniewska, Tomasz Urbanowicz, Andrzej Jawień, Zbigniew Krasiński, Dorota Formanowicz

**Affiliations:** 1 Department of Medical Chemistry and Laboratory Medicine, Poznan University of Medical Sciences, Rokietnicka 8, 60-806 Poznan, Poland; 2 Department of Cardiac Surgery and Transplantology, Poznan University of Medical Sciences, 61-701 Poznan, Poland; 3 Department of Vascular and Endovascular Surgery Angiology and Phlebology, Poznan University of Medical Sciences, 61-701 Poznan, Poland; 4 Department of Stem Cells and Regenerative Medicine, Institute of Natural Fibres and Medicinal Plants-National Research, Kolejowa 2, 62-064 Plewiska, Poland

**Keywords:** antioxidants, aortic aneurism, NRF2, bilirubin, PON1, HO-1, redox status, reductive stress

## Abstract

The progression of an abdominal aortic aneurysm (AAA) is an important issue, especially as AAA is becoming more common, and potentially life-threatening. This study aimed to understand better the mechanisms underlying AAA progression. For this purpose, we have focused on assessing the selected biomarkers whose potentially common denominator is the NRF2 (nuclear factor erythroid 2-related factor 2) transcription factor, that determines the selected antioxidant enzymes’ activation. The study group consisted of 44 AAA male patients (71.41 ± 7.80 years aged). They were divided into three groups based on the aneurism diameter: group I (below 55 mm), group II (between 55 and 70 mm), and group III (over 70 mm). The laboratory analyses of PON1 (paraoxonase-1), NRF2, and HO-1 (heme oxygenase 1) were performed based on commercial ELISA tests; Blb (bilirubin) and hsCRP (high sensitivity C-reactive protein) were assessed during routine morphology examinations after admission to the hospital. Multiple linear regression showed that both bilirubin and NRF2 determined the PON1 concentration in the entire study group. The correlations between the examined parameters within the three studied groups suggest the capitulation of NRF2-dependent antioxidant mechanisms to pro-inflammatory processes. We showed that HO-1 and hsCRP may play a crucial role in the development of inflammation aneurism progression. Moreover, in patients with medium-sized aneurysms, antioxidant mechanisms were depressed, and inflammatory processes began to dominate, which may lead to uncontrolled growth aneurysm rupture. Our study is one of the first to indicate that the chronically activated antioxidant pathway using NRF2 may be a source of reduction stress.

## 1. Introduction

Abdominal aortic aneurism (AAA), defined as the abdominal aorta maximal transverse diameter exceeding the physiologic diameter immediately above the dilation by at least 50%, is usually degenerative and often associated with atherosclerosis [1,2]. Currently, surveillance or interventional strategy is recommended depending on the patient’s profile. AAA is often asymptotic, and its silent character is the main cause of late diagnosis. Limet et al. [3] showed that the progression in AAA size increases the risk of rupture. As the disease progresses, the initiation of enzymatic processes tends to accelerate the destruction of collagen, hence, the aneurysm’s enlargement [3,4]. Several risk factors for aneurysm progression and rupture are common with coronary artery disease, including diabetes, smoking, and arterial hypertension. Since inflammation has been postulated as a crucial factor in atherosclerosis, AAA may also have an inflammatory background [4,5,6]. However, molecular pathomechanisms of AAA formation and progression are still unclear. Several markers have been reported as significant in AAA progression [7,8]. Increased white blood cell count and decreased bilirubin levels are correlated with AAA progression [9]. Bilirubin (Blb) has antioxidant and anti-inflammatory features, which have been documented in patients with Gilbert’s syndrome [10,11]. The analysis of male patients with cardiovascular disease showed a significant correlation between decreased bilirubin concentration and increased risk of atherosclerosis [11]. The data in recent years strongly suggest that bilirubin behaves as a signaling molecule—affecting various cell regulatory systems and binding to multiple cell membranes and nuclear receptors. It was demonstrated that bilirubin mediates the up-regulation of several nuclear factors including erythroid 2-related factor 2 (NRF2) subjects via Antioxidant Response Element (ARE) induction [12,13,14].

The moderate oxidative stress induces NRF2 to leave its inhibitor Keap1 (Kelch-like ECH-associated protein 1) and translocate into the nucleus. Subsequently, NRF2 binds to the ARE present in the deoxyribonucleic acid (DNA) sequence of a broad group of antioxidant compounds, including paraoxonase-1 enzyme (PON1), and heme oxygenase 1 (HO-1), and induces their transcription [15,16,17,18]. PON1 is a high-density lipoprotein (HDL)-associated enzyme with antioxidant potential, preventing low-density lipoprotein (LDL) oxidation by phospholipase A2-like activity towards oxidatively modified phospholipids. As a result, PON1 inhibits the differentiation of monocytes into macrophages, which in turn maintains vascular smooth muscle cells (VSMC) density and elastin structure. Decreased circulating PON1 was found to be associated with AAA in human and mice models [19].

Although oxidative stress and inflammation’s contribution to aneurysm pathophysiology has been underlined in recent years, there are still many uncertainties, particularly in the issue of AAA progression. The average rate of aneurysm growth covers a wide range of variability and is difficult to predict. The introduction of optimal biomarkers for diagnosing aneurysm progression is of the highest value.

We hypothesized that a high Blb level triggers the NRF2 for transcripting PON1. Therefore, the current study aimed to analyze potential initiators, carriers of information, and biomarkers included in the process of aneurysm progression. The study was designed to observe the interactions between parameters known for its antioxidant/redox potential.

## 2. Materials and Methods

### 2.1. Material

The study was performed in a group of 44 AAA male patients (71.41 ± 7.80 years aged) who were admitted to the Department of Vascular, Endovascular Surgery, Angiology and Phlebology, University of Medical Sciences, Poznan, Poland, for surgery. The exclusion criteria were female sex, diabetes mellitus, and active smoking or in the last 10 years. Arterial hypertension and dyslipidaemia were diagnosed in all enrolled patients. All study participants underwent Doppler ultrasonography, computed tomography (CT), and/or arteriography before surgery. The maximal abdominal aorta diameter was measured by reviewing each coronal CT section. We considered the use of statins and ACEI, but we did not notice any statistical significance. The study group was divided into 3 groups according to the size of the aneurysm: group I (below 55 mm), group II (between 55 and 70 mm), and group III (over 70 mm). All subjects gave their informed written consent for inclusion before participating in the study. The study was conducted in accordance with the Declaration of Helsinki, and the Ethics Committee of Poznan University of Medical Science approved the protocol (number 854/14). Blood samples for blood morphology and biochemistry analyses were collected at the admission before the surgery. Glomerular filtration rate (GFR) was calculated by the simplified Modification of Diet in Renal Disease (MDRD) formula. The blood samples for analysis were collected into lithium heparin tubes before surgery and after centrifugation, were aliquoted, and frozen at −80 °C until analyzed. Demographic and clinical data were obtained and analyzed.

### 2.2. Methods

Blood morphology was evaluated using a routine hematology analyzer (Sysmex Europe GmbH, Norderstedt, Germany).

The unconjugated bilirubin concentration assay procedure has been validated and standardized to avoid laboratory errors and ensure repeatability of results. The determination was performed on the Atellica CH 930 biochemical analyzer, Siemens Healthcare Diagnostics Inc. Laboratory Diagnostics 511 Benedict Avenue Tarrytown, NY 10591-5005, USA.

NRF2 was measured by the commercially available RayBio Human NRF2 ELISA KIT (Ray Biotech Life, Peachtree Corners, GA, USA). Briefly, standards and samples were pipetted into the antibody specific for human NRF2-coated wells. NRF2 presented in a sample was bound to the wells by the immobilized antibody. After following steps, including washing unbound antibody, pipetting the HRP-conjugated streptavidin, and adding TMB substrate, the development of blue colour was observed in proportion to the amount of NRF2 bound. The stop solution changed the blue colour to yellow, and the absorbance was measured at 450 nm.

PON1 was measured following the protocol provided by the ABNOVA PON1 (Human) ELISA Kit. Briefly, the reaction is based on a solid phase immunoassay; after adding the standards and the samples to the wells, the biotinylated antibody was added. Following the procedure, the absorbance of the yellow product was measured at 450 nm, and the absorbance of the product was linearly proportional to the Human PON1 concentration in the sample.

HO-1 was measured following the protocol provided by the ABCAM Elisa Kit. The assay employs the affinity tag labeled capture antibody and a reporter conjugated detector antibody, which immunocapture the sample analyte in solution. This complex was immobilized via immunoaffinity coating the wells. Briefly, the standards and samples were added to the wells, followed by the antibody mix. Following the procedure, the absorbance of the yellow product was measured at 450 nm, and the absorbance of the product was linearly proportional to the Human HO-1 concentration in the sample.

Serum iron concentration was measured following the protocol provided by a commercially available Biomaxima kit (Biomaxima, Lublin, Poland). Briefly, serum iron ions bound to transferrin were released by guanidine hydrochloride, reduced to ferrous ions, and subsequently complexed by Ferrosyn to form a coloured product with an absorbance maximum at 560 nm.

### 2.3. Statistical Analyses

Data were statistically analyzed using Graph Pad Prism Version 9.5.1 (GraphPad Software, 128 San Diego, CA, USA). Data were presented as mean ± SD (standard deviation) when they passed the normality test, and median and min-max range if they did not meet the Gaussian distribution criteria. The D’Agostino-Pearson normality test was used to test the normality of the data. One-way ANOVA was used to compare parameters between the study groups, the correlation coefficients were presented in a matrix, and the *p*-value type computed by Prism is Spearman. Categorical variables were analyzed with the Chi-test, while multiple linear regression was used to assess the dependence of the influence of the tested parameters. *p* ≥ 0.05 was considered statistically significant.

## 3. Results

### 3.1. Comparisons

The analyses of demographic and routine laboratory parameter assessments revealed some statistically significant differences regarding the aneurysm size; see Table 1. PLT, GFR, and fibrinogen were significantly lower in group II than in groups I or III. In contrast, creatinine and urea in group II were significantly higher than in groups I or III. hsCRP and WBC significantly increased with the size of the aneurysm, while HDL showed the opposite changes.

In Table 2 studied oxidative stress—related biomarker characteristics is presented.

**Figure 1:**
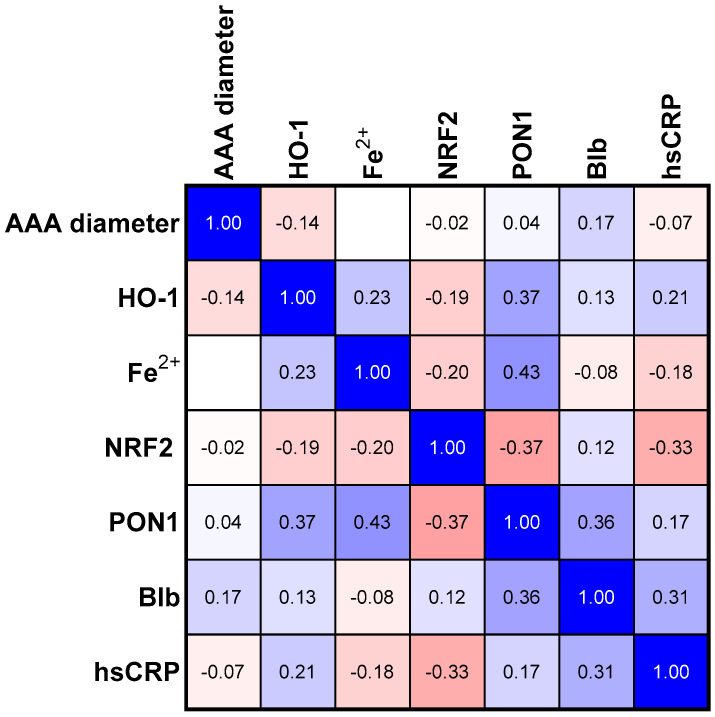
Correlation matrix (r coefficient) for the analyzed parameters performed for the entire study group. Abbreviations: HO-1—heme oxygenase; NFR2—nuclear factor erythroid 2—related factor 2, PON1—paraoxonase—1, Blb—bilirubin, hsCRP—high sensitivity C—reactive protein.

**Figure 2:**
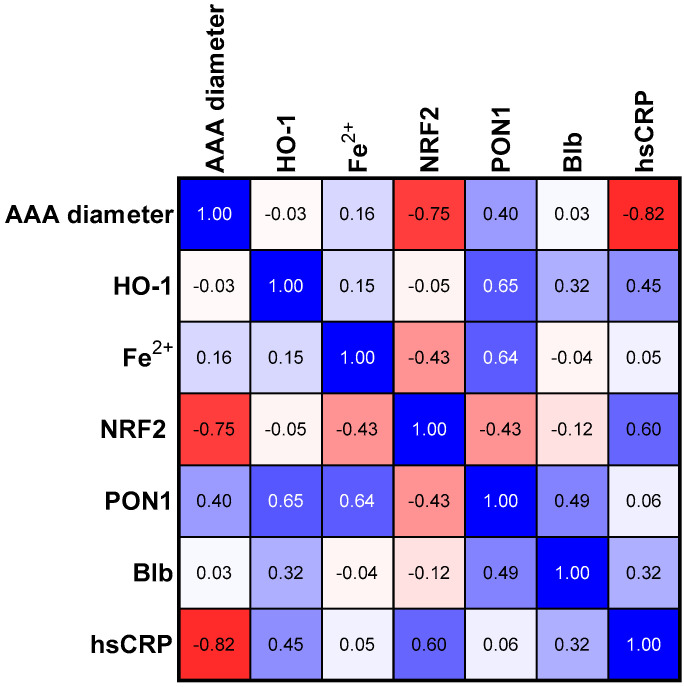
Correlation matrix (r coefficient) in group I with aneurism diameter size below 55 mm. Abbreviations: HO-1—heme oxygenase; NFR2—nuclear factor erythroid 2—related factor 2, PON1—paraoxonase—1, Blb—bilirubin, hsCRP—high sensitivity C—reactive protein.

**Figure 3:**
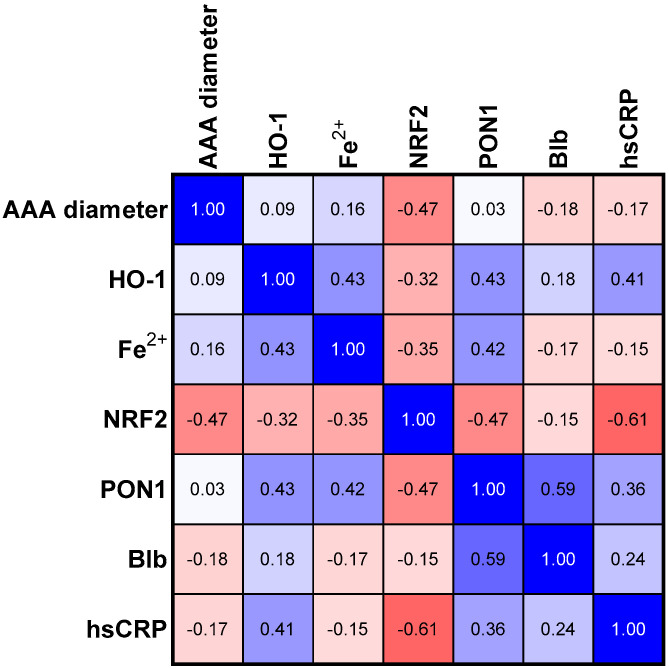
Correlation matrix (r coefficient) in group II with aneurism diameter size between 55–70 mm. Abbreviations: HO-1—heme oxygenase; NFR2—nuclear factor erythroid 2-related factor 2, PON1—paraoxonase—1, Blb—bilirubin, hsCRP—high sensitivity C—reactive protein.

**Figure 4:**
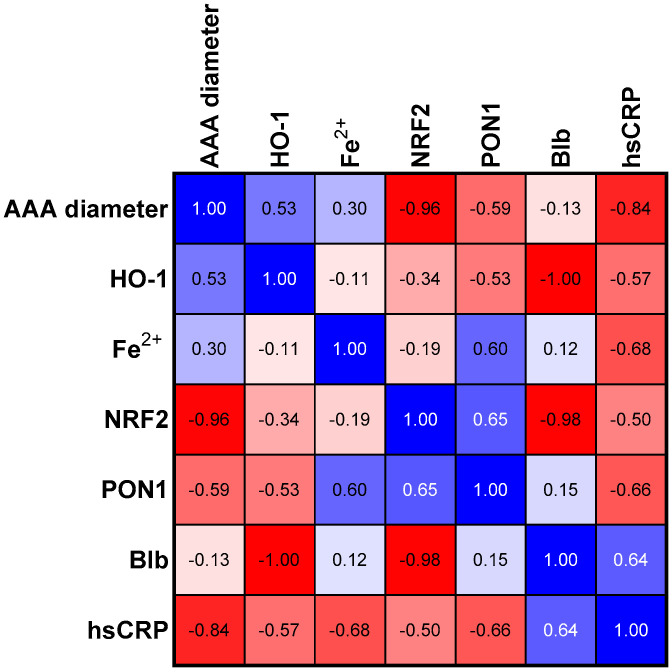
Correlation matrix (r coefficient) in group II with aneurism diameter size above 70 mm. Abbreviations: HO-1—heme oxygenase; NFR2—nuclear factor erythroid 2-related factor 2, PON1—paraoxonase—1, Blb—bilirubin, hsCRP—high sensitivity C—reactive protein.

The analyses of PON1, bilirubin, and NRF2 concentrations in three studied groups revealed a common trend for these parameters (Table 2). Bilirubin and NRF2 in the group with the average size of the aneurysm (Group II) showed a statistically significant higher concentration than in Group I. The group of patients with the most advanced aneurysm (Group III) was characterized by a decrease in the concentration of the three analyzed parameters mentioned above if compared to Group II. The concentration of PON1 and HO-1 was the highest in Group II compared to the other groups; however, in both cases, the tendency was not significant (Table 2). In the next step, we assessed the relationships between the selected parameters in the study group (Figure 1) and in groups categorized according to the aneurysm size (Figure 2, Figure 3 and Figure 4).

### 3.2. Correlations

The correlation matrix analysis of parameters in the study group, without categorization into the stage of aneurysm, shows a number of correlations in which the PON1 concentration plays a key role (Table 3, Figure 1). In this group, we observed a negative correlation between the concentrations of PON1 and NRF2 and a negative correlation between the concentrations of hsCRP and NRF2. We also observed a positive correlation between bilirubin and hsCRP.

Positive correlations between PON1 concentration with HO-1, Fe^2+^, and bilirubin concentrations were found (Table 3). The categorization of the study group in terms of the size of the aneurysm made it possible to analyze the progression of the aneurysm diameter in light of changes in the examined parameters. In the next step, we conducted in-depth analyses of the parameters studied in individual groups in terms of the size of the aneurysm.

### 3.3. Correlations in SMALL Size Aneurism Group

Two statistically significant negative correlations between the diameter of the aneurysm in the group of patients with the smallest aneurysm and the concentration of the transcription factor NRF2 and hsCRP turned out to be interesting (Table 3, Figure 2). A trend towards a significant positive correlation between PON1 and aneurysm diameter was observed. The negative but not significant correlation of NRF2 with the concentration of PON1 and the concentration of Fe^2+^ ions was also surprising. In this group, we also saw a positive interaction between NRF2 and hsCRP concentrations. As expected, we found a positive correlation between PON1 concentration and individual parameters, like HO-1, bilirubin, and Fe^2+^ ion concentration.

### 3.4. Correlations in Medium Size Aneurism Group

In the group of patients with medium aneurysm size, there was still a significant negative correlation between the aneurysm diameter and NRF2 concentration. However, the nature of the previously described significant correlation between NRF2 and hsCRP concentrations changed from positive to negative. In this group, the correlations of PON1 concentrations with HO-1 and Fe^2+^ weakened, while the positive correlation between PON1 and bilirubin concentrations strengthened. There was also a positive, although not statistically significant, trend between PON1 and hsCRP concentrations. The trend towards a negative correlation between NRF2 and PON1 concentration became statistically significant. (Table 3, Figure 3).

### 3.5. Correlations in Large Size Aneurism Group

Within the group with the most advanced aneurysm size, there was a weak significant correlation between HO-1 concentration and the size of the aneurysm. The inverse correlation between aneurysm size and NRF2 concentration became extremely strong. The inverse correlation between NRF2 and hsCRP concentrations still persisted, but lost its significance. As in group I, there was a strong inverse correlation again between hsCRP concentration and aneurysm size. Two new strong interactions also appeared in this group. The first was a positive correlation between PON1 and NRF2 concentrations, and the second was an inverse correlation between PON1 and hsCRP concentrations. We observed two powerful inverse interactions for the first time among all groups. First, between HO-1 and bilirubin, and second, bilirubin with NRF2 concentration (Table 3, Figure 4). We suspected that the strength of these correlations weakened the statistical significance of other interactions present in this study group.

Multivariate linear regression was performed to understand how changes in the variables studied contribute to explaining the PON1 concentration as a result of interest in this study.

Consequently, the predictive value for PON1 concentration in the study group was observed in the case of bilirubin and NRF2 concentrations (Table 4).

### 3.6. Contingency Table for Bilirubin Cut-Off Values

**Figure 5:**
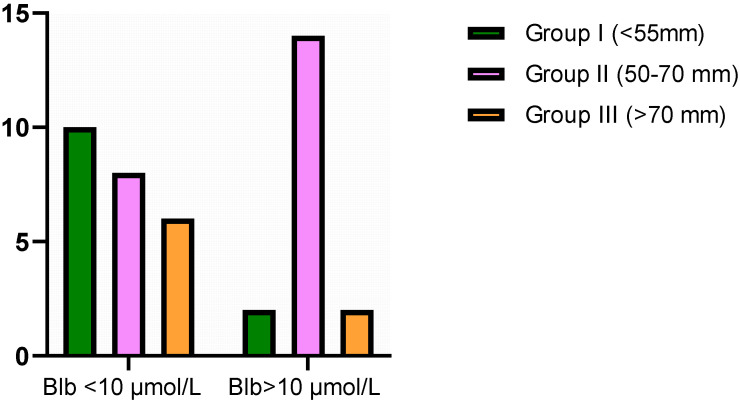
Graphical representation of the distribution of Blb (bilirubin) values above and below 10 μmol/L according to the aneurysm size. Statistical significance based on Chi –square test *p* = 0.0159.

To assess whether the discrepancy between the incidence of Blb values above or below 10 μmol/L in the studied groups (different in terms of aneurysm size) was more than expected by chance, a Chi –square test was performed (Figure 5).

The group with the most patients with bilirubin above the cut-off value was the group with the average size of the aneurysm. Surprisingly, the largest proportion of patients with bilirubin below 10 μmol/L belonged to the group with the smallest aneurysm size.

## 4. Discussion

For the first time, we analyzed the cross-talk between bilirubin, NRF2, and PON1 in accordance with the progression of the aneurysm. Therefore, we expected to observe a positive correlation between Blb and PON1. NRF2 may be a link/messenger between Blb and PON1, regulating the redox potential of the body. Because of higher Blb, and PON1 concentrations, we expected to reveal an aneurism size impact. The HO-1 activity is the main source of bilirubin in cells, and it is also induced via the ARE signaling pathway by NRF2 [20]. The role of HO-1 is, however, multiple. It can be perceived as a potential antioxidant, being a source of bilirubin, and on the other hand, it is also a source of free iron ions, acting pro-oxidatively in the Fenton reaction, and the powerful reducing agent, heme. Moreover, it can activate tumor necrosis factor α (TNF-α) transcription [21] by stimulating the nuclear factor kappa-light-chain-enhancer of activated B cells (NF-κβ). The results of our study indicate that the role of HO-1 in the process of aneurysm progression may be ambiguous, with an indication of a pro-inflammatory one. This is suggested by tendencies towards a positive correlation between HO-1 and hsCRP, both in group I and II, and is in line with the results of the Hofmann study [17]. In the group with the most advanced aneurysm, the concentration of HO-1 positively correlates with the diameter of the aneurysm, while surprisingly remaining inversely correlated with the bilirubin concentration. Such behavior of HO-1 may indicate its increasing participation with the progression of the disease in the processes leading to the degradation of vascular wall cells.

The results of our research suggest that during the progression of the aneurysm, and especially in the transitional stage in the group of patients with aneurysm diameter between 55 and 70 mm, the oxidative stress increases to such an extent that chronic activation of NRF2 is a source of redox homeostasis disturbance of cells, intensifying inflammatory processes and leading to non-linear development of the aneurysm, which may potentially lead to its rupture.

As the aneurysm grows, antioxidant mechanisms are mobilized (particularly seen in the parameters measured, especially in group II) and may progress to the processes leading to cell degradation and apoptosis. Changes in the course of lipid parameters and PLT, fibrinogen, or GFR in the study groups are observed in patients with atherosclerosis treated in our clinic. Hence, it can be concluded that the atherosclerotic process coexists with the progression of the aneurysm. Analysis of the clinical data for aneurysm size in Table 1 indicates a disturbance in homeostasis of studied patients. This is also depicted by hsCRP concentration, showing increasing inflammation consistently parallel with increasing aneurysm size. Many groups have studied the involvement of hsCRP in inflammation; it is known to play a key role in promoting atherothrombosis by affecting endothelial cells, endothelial progenitor cells, monocyte-macrophages transformation, and smooth muscle cells [22,23]. Moreover, in vascular smooth muscle cells, hsCRP has been shown to stimulate the pro-inflammatory NF-κB pathway [24]. In the inflammatory process leading to the development of an abdominal aortic aneurysm, T-cells and macrophages are known to be the dominant markers of aneurysm tissue [4]. According to our analysis, the process changes with the increase in aortic aneurysm diameter—first acting as a compensative mechanism and with a larger diameter—losing balance in the compensative potential and, thus, forwarding into a severely devastating mechanism. Inflammatory activation is a defensive mechanism vital for health, and aims to regain disturbed hemostasis. It triggers reactions that play a significant role in healing processes, but may also result in artery wall structural derangements by responsive mechanisms triggered by oxidative stress. Chronic inflammation in AAA patients during the course of the disease, together with oxidative stress, may lead to excessive heme accumulation due to the action of HO-1. Due to its reductive character under such conditions, heme may favor and intensify pathomechanisms leading to leukocyte activation and migration, up-regulation of adhesion molecules, and induction of cytokines and acute phase proteins. Heme has been shown to induce the secretion of TNF-α by macrophages dependently of myeloid differentiation markerMyD88, toll-like receptor 4 (TLR4), and cluster of differentiation 14 (CD14) [21,25]. In turn, research published by Batra indicates that it is TNF-α that is highly responsible for the development of aneurysms [5]. The results of our research, analyzing selected parameters of oxidative stress and pro-inflammatory factors, may improve the knowledge in the already known mechanisms, and bring new suggestions for potential therapeutic strategies.

The vector of the analyzed interaction reveals the role of NRF2 as a transmitter of information between HO-1, bilirubin, and PON1, i.e., the parameters whose participation in the development of an aneurysm has been described as potentially significant. The multivariate linear regression revealed that bilirubin and NRF2 were independent predictors of PON1 concentration in the study group. The continuing trend of increasing PON1 concentration with increasing aneurysm size may also refer to its role as an acute phase protein, which has already been described in our previous work [26]. Studies by Burillo et al. indicate that the concentration of PON1 in patients with AAA is 2.5 times lower than in the control group [19]. The analysis of individual study groups, which we propose, considering the aneurysm size, allows us to observe the significance of PON1 concentration. The trend of increase in the concentration of PON1, though statistically not significant, was observed in all patients group, and may be interpreted as an antioxidant response of the body to increasingly intensifying oxidative stress, which corresponds with an increase in the concentration of NRF2 and bilirubin.

Previous studies [11] indicated a total plasma bilirubin cut-off value of 10 μmol/L. The algorithm described by the authors suggested that each micromolar decrease in total bilirubin significantly increases the risk of cardiovascular disease. Our results indicate that the highest percentage of patients with total bilirubin below the cut-off value presented by Gazzin was observed in the plasma of patients with small-sized aneurysms. Meanwhile, the medium aneurysm group had the highest percentage of patients with bilirubin below 10 μmol/L. The results of our studies in this aspect reveal the occurrence of the aneurysm (group I) due to the high risk of CVD associated with low bilirubin concentration, while group II (medium size) illustrates the critical mobilization of antioxidant mechanisms with the participation of bilirubin. Our study also points to the consistent, stable nature of bilirubin, especially in the group with the largest aneurysm, as it is the only antioxidant parameter negatively correlating with the dominant hsCRP in this analysis.

We aimed to test the hypothesis based on available, uncomplicated, and quick results methods; hence, the determinations were made using the methods described above, and the material for the study was the patients’ plasma.

In recent years, the existence of an interplay between NF-kB and Nrf2 signaling has been described, while indicating that this complex relationship can enhance or weaken the inflammatory response, depending on the specific context and stimuli [27,28,29]. Our research is in line with the conclusion presented by Bellezza’s study, that chronic activation of the NRF2-mediated pathway has deleterious consequences for organ functions [15]. They result from the imbalance between oxidation and reduction processes, and the constantly active path fighting reactive oxygen species and free radicals leads to an increase in the amount of reducing substances. Reductive stress is consequently just as harmful as oxidative stress.

The results of our research, although preliminary and requiring a deeper analysis, raise doubt on the therapeutic increase in the activity of the antioxidant path associated with pharmacological NRF2 activation. Capturing the critical moment before the capitulation of antioxidant mechanisms would possibly be a chance to introduce effective therapy, leading to better control over the unpredictable rate of aneurysm growth. At the present stage, it seems crucial to identify direct and indirect targets induced by NRF2, as well as their possible involvement in pro-inflammatory mechanisms, such as the NFκB pathway. Therefore, analyzing the interpenetrating mechanisms, both pro- and antioxidant, is justified.

### Limitations of the Study

The limitation is the relatively small group of patients included in the study. In addition, it should be emphasized that we did not focus on the patient’s condition, method of treatment, and postoperative course. Our study focused on pathophysiological processes in the aneurysmal aortic wall within its diameter progression. Therefore, we decided that the analysis of the parameters selected for the study would not include the control group (healthy population), and we would make comparisons within the groups divided by the severity of the disease.

## 5. Conclusions

Our research suggests that during aneurysm progression, chronic activation of the NRF2 pathway dysregulates the redox balance towards reductive stress.

Antioxidant mechanisms in the group with an aneurysm diameter above 70 mm are dominated by pro-inflammatory mechanisms, leading to the degradation of the vascular wall cells, which results in uncontrolled progression of the aneurysm and rupture.

HO-1 may play a role in both defense and pro-inflammatory mechanisms leading to aneurysm progression.

## Figures and Tables

**Table 1:** Demographic and routine laboratory parameter characteristics in studied groups. Continuous variables are expressed either as the medians (min-max) or means (SD), depending on normality test results—* statistically significant with *p* ≥ 0.05. Abbreviations: ns—not significant; BMI—body mass index, GFR—glomerular filtration rate, HDL cholesterol—high-density lipoprotein cholesterol, hsCRP—high sensitivity C-reactive protein, LDL cholesterol—low−density lipoprotein cholesterol, PLT—platelet count, RBC—red blood cell count, TG—triglycerides, WBC—white blood cell count, n—number of patients.

	Group I (n = 12)	Group II (n = 22)	Group III (n = 10)	ANOVA *p*-Value
Aneurism size (mm)	50.00 (34.00–55.00)	66.00 (58.00–70.00)	75.00 (74.00–85.00)	<0.0001
Age	72.17 ± 3.512	74.64 ± 8.18	62.25 ± 4.559	<0.001 *
BMI	29.93 ± 2.935	27.19 ± 4.988	25.12 ± 4.522	ns
LDL cholesterol [mmol/L]	2.57±2.08	1.982±0.8	2.775 ± 0.462	ns
HDL cholesterol [mmol/L]	1.751 ± 0.8	1.291 ± 0.43	1.305 (0.770–1.580)	0.031 *
Total cholesterol [mmol/L]	4.410 (2.280–10.23)	3.878 ± 0.906	4.598 ± 0.369	ns
TG [mmol/L]	1.362 ± 0.65	1.286 ± 0.64	1.353 ± 0.432	ns
Glucose [mmol/L]	6.077 ± 1.39	6.206 ± 0.857	7.440 ± 1.483	ns
hsCRP [mg/L]	9.25 ± 6.86	10.37 ± 5.542	12.61 ± 9.891	ns
WBC [10^9^/L]	6.486 ± 0.923	7.030 ± 2.751	9.555 ± 3.817	0.023 *
RBC [10^9^/L]	4.555 ± 0.29	4.540 (3.990–5.290)	4.458 ± 0.149	ns
HGB [mmol/L]	8.162 ± 0.614	8.609 ± 1.081	8.350 ± 0.469	ns
PLT [10^9^/L]	245.3 ± 60.68	204.5 ± 75.43	400.5 ± 175.1	<0.001 *
GFR [ml/min/1.73 m^2^]	73.50 ± 7.46	62.00 (16.00–72.00)	76.65 ± 5.24	<0.001 *
Creatinine [μmol/L]	87.19 ± 18.74	102.4 (76.50–363.0)	83.83 ± 12.30	0.043 *
Fibrinogen [mg/dL]	281.0 (270.8–436.5)	287.3 ± 81.61	394.9 ± 136.1	0.021 *
Urea [mmol/L]	6.462 ± 2.467	7250 (3.950–21.12)	4.255 ± 2.034	0.038 *

**Table 2:** Studied oxidative stress—related biomarker characteristics. Continuous variables are expressed as medians (min-max) or means (SD); *—statistically significant with *p* ≥ 0.05. Abbreviations: ns—not significant; HO-1—heme oxygenase; NFR2—nuclear factor erythroid 2-related factor 2, PON1—paraoxonase-1, Blb—bilirubin, n—number of patients.

	Group I (n = 12)	Group II (n = 22)	Group III (n = 10)	ANOVA *p*-Value
HO-1 [ng/mL]	3.64 ± 1.56	3.74 ± 1.65	2.469 ± 1.146	ns
Fe^2+^ [μmol/L]	10.01 ± 6.13	9.46 ± 3.57	9.867 ± 4.314	ns
NRF2 [ng/mL]	1.37 (0.67–2.2)	3.48 (0.8404–8.58)	2.299 (1.466–2.48)	0.032 *
PON1 [ng/mL]	328.84 ± 147.9	367.6 ± 321.8	409 ± 153.7	ns
Blb [μmol/L]	8.41 ± 1.89	12.29 ± 3.89	8.90 ± 4.351	0.007 *

**Table 3:** Study of correlations occurring within the study groups categorized in terms of the size of the aneurysm and the entire group. The table presents numerical values of statistically significant correlations. Abbreviations: ns—not statistically significant; AAA—aneurism diameter, HO-1—heme oxygenase; NFR2—nuclear factor erythroid 2—related factor 2, PON1—paraoxonase—1, Blb—bilirubin, hsCRP—high sensitivity C—reactive protein.

*p* Values	Group I (<55 nm)	Group II (55–70 nm)	Group III (>70 nm)	AAA Group
AAA vs. NRF2	0.007	0.049	<0.001	ns
AAA vs. hsCRP	0.001	ns	0.035	ns
PON1 vs. HO-1	0.015	0.061	ns	0.018
PON1 vs. NRF2	ns	0.049	0.079	0.023
PON1 vs. Fe^2+^	0.018	0.066	ns	0.005
PON1 vs. Blb	ns	0.006	ns	0.023
HO-1 vs. Blb	ns	ns	<0.001	ns
HO-1 vs. Fe^2+^	ns	0.047	ns	ns
hsCRP vs. Blb	ns	ns	ns	0.046
hsCRP vs. NRF2	0.05	0.008	ns	0.056
Blb vs. NRF2	ns	ns	<0.001	ns

**Table 4:** Multivariate linear regression model with PON1 concentration as the dependent variable in the study group, without division into the stage of aneurysm. * statistically significant. Abbreviations: HO-1—heme oxygenase; NFR2—nuclear factor erythroid 2—related factor 2, Blb—bilirubin.

Variable	95% CI	*p*-Value
Intercept	−621.1 to 302.0	0.485
AAA diameter	−5.435 to 7.523	0.744
HO-1	−9.522 to 64.54	0.140
Fe^2+^	−5.510 to 31.61	0.161
NRF2	−29.27 to −1.934	0.027 *
Blb	9.591 to 48.42	0.005 *

## Data Availability

All necessary data are included in the paper.
